# *Fasciola hepatica* Excretory-Secretory Products (*Fh*-ES) Either Do Not Affect miRNA Expression Profile in THP-1 Macrophages or the Changes Are Undetectable by a Microarray Technique

**DOI:** 10.3390/pathogens13100854

**Published:** 2024-10-01

**Authors:** Piotr Bąska, Alicja Majewska, Wojciech Zygner, Ewa Długosz, Marcin Wiśniewski

**Affiliations:** 1 Division of Pharmacology and Toxicology, Department of Preclinical Sciences, Institute of Veterinary Medicine, Warsaw University of Life Sciences, 02-786 Warsaw, Poland; 2 Department of Physiological Sciences, Institute of Veterinary Medicine, Warsaw University of Life Sciences (SGGW), Nowoursynowska 159b, 02-776 Warsaw, Poland; alicja_majewska@sggw.edu.pl; 3 Division of Parasitology and Parasitic Diseases, Department of Preclinical Sciences, Institute of Veterinary Medicine, Warsaw University of Life Sciences, 02-786 Warsaw, Poland; wojciech_zygner@sggw.edu.pl (W.Z.); ewa_dlugosz@sggw.edu.pl (E.D.); marcin_wisniewski@sggw.edu.pl (M.W.)

**Keywords:** *Fasciola hepatica*, liver fluke, immune response, miRNA, microarray

## Abstract

*Fasciola hepatica* is a liver fluke that resides in the bile ducts of various mammals. The parasitosis leads to economic losses in animal production estimated at USD 3.2 billion annually. It is also considered a zoonosis of great significance and a problem for public health affecting 2.4 million people worldwide. Nevertheless, besides the negative aspects of infestation, the antigens released by the fluke, *F. hepatica* Excretory-Secretory Products (*Fh*-ES) contain several immunomodulatory molecules that may be beneficial during the course of type I diabetes, multiple sclerosis, ulcerative colitis, or septic shock. This phenomenon is based on the natural abilities of adult *F. hepatica* to suppress proinflammatory responses. To underline the molecular basis of these mechanisms and determine the role of microRNA (miRNA) in the process, lipopolysaccharide (LPS)-activated THP-1 macrophages were stimulated with *Fh*-ES, followed by miRNA microarray analyses. Surprisingly, no results indicating changes in the miRNA expression profile were noted (*p* < 0.05). We discuss potential reasons for these results, which may be due to insufficient sensitivity to detect slight changes in miRNA expression or the possibility that these changes are not regulated by miRNA. Despite the negative data, this work may contribute to the future planning of experiments by other researchers.

## 1. Introduction

*Fasciola hepatica* is a liver fluke found worldwide, infesting both wild and domestic animals, as well as humans. It is considered the most significant cause of cattle helminthiasis in Africa, with a prevalence of 30 to 90% [1], whereas in Australia, 46% of herds are likely to experience fluke-related production losses [2]. In Europe, it is becoming a re-emerging sheep disease, with infestation rates ranging from 6% to 61% [3]. Infested livestock suffer from severe anemia, liver failure, increased susceptibility to secondary infections, and, in cases of heavy infestations, death may occur [4]. This results in a reduction in milk yield, diminished weight gain, and decreased fertility, resulting in global economic losses estimated at USD 3.2 billion per year [5]. Humans can be accidentally infested by consuming water or plants contaminated with metacercariae, which is common for rural regions with low hygiene standards in the endemic areas. Infested individuals may suffer from right upper quadrant discomfort, anorexia, acute cholecystitis, and biliary obstruction; nevertheless, in most cases, the parasitosis is asymptomatic. Estimates indicate that 2.4 million people suffer from the disease, and a further 180 million are at risk, highlighting fascioliasis as a significant threat to public health. The scale of the problem led the World Health Organization (WHO) to classify human fascioliasis as a foodborne zoonosis belonging to the Neglected Tropical Diseases [6]. The Disability-Adjusted Life Years (DALY) value for the disease is estimated at 35,000, meaning that 35,000 years of human health are lost annually due to prevalent cases in the human population [7]. The presence of parasites in the bile ducts during the chronic phase is often asymptomatic, allowing the fluke to reproduce. Adult flukes release unembryonated eggs into the host’s bile ducts, which are then passed into the environment with stool during defecation. In water, the eggs become embryonated and hatch into free-swimming, ciliated miracidia that seek an intermediate host, typically a snail from the Lymnaeidae family [8]. Inside the snail’s tissues, the miracidia undergo several asexual metamorphoses, starting as sporocysts, which then transform into rediae, giving rise to another generation of rediae through clonal expansion [7,9]. Finally, the redia develops into a free-living cercaria, which exits the snail into the water [10], followed by encystation into metacercaria, which awaits ingestion by the definitive host. Once in the host’s duodenum, the metacercaria excysts into the newly excysted juvenile developmental stage (NEJ) and migrates through the intestinal wall and the peritoneum [11]—a process associated with the prehepatic (or the early) stage. Upon reaching the liver capsule, the immune system reacts vigorously, which is associated with the acute stage, manifesting as tissue destruction, inflammation, local or systemic toxic/allergic reactions, and internal damage [12]. The acute phase ceases once the parasite reaches the bile ducts, and the chronic phase begins, which may last 5 to 8 years [12]. The symptoms may resemble those of the acute stage but are usually more discrete [12]. Although fever, malaise, abdominal pain, eosinophilia, hepatomegaly, nausea, weight loss, liver failure [2,13], and biliary duct obstruction may occur [3,14], this stage is often asymptomatic.

During viral and bacterial infections or parasitoses, the type of immune response depends on a complicated interplay between the host and the intruder. It can be divided into four main subtypes: Th_1_, Th_2_, Th_17_, and T_reg_ [15], although sometimes Th_1_ and Th_17_ responses are classified as Th_1_/Th_17_ due to overlapping effects [16]. These names refer to T helper (Th) and regulatory T (T_reg_) cell populations that orchestrate the particular response. Activated Th_1_ cells release significant amounts of interferon gamma (IFN-γ) and IL-12, activating cellular and inflammatory mechanisms, including proinflammatory M1 macrophages involved in phagocytosis and cell-mediated immunity, which are engaged in fighting intracellular pathogens and induce tissue damage. During a Th_17_ response, Th_17_ cells release substantial amounts of IL-17 and IL-22, which activate mechanisms against extracellular bacteria and fungi [17]. Th_2_ cells release IL-4, IL-5, and IL-13, leading to the alternative activation of macrophages, which counteract inflammation and promote tissue repair [18]. T_reg_ (regulatory T cells) release IL-10 and transforming growth factor beta (TGF-β), which mitigate the effects of the other responses [19]. All of these types of immune responses need to be balanced and strictly regulated. Failure to control these processes can result in allergies, autoimmune diseases, or immunosuppression. Infectious agents elicit various immune responses depending on the pathogen, its isolate/strain [11,20], and the host’s genetics [21]. During the acute phase of fascioliasis, the predominant response is a Th_2_/Th_17_ response, which contributes to the liver fibrosis [11]. Once the parasite settles in the bile ducts, the immune response shifts towards T_reg_ [11], which is beneficial for both the host and the parasite. The host avoids immunopathology, while the parasite can survive and reproduce. It is in the parasite’s interest to modulate the immune response, so it releases a number of bioactive molecules in *Fasciola hepatica* Excretory-Secretory Products (*Fh*-ES), which contain antioxidants, fatty acid binding proteins (FABPs), cysteine proteases, protease inhibitors, mucin-like peptides, TGF-β mimicking molecules, helminth defense molecules (HDMs) [22], as well as extracellular vesicles containing immunomodulatory proteins [23,24,25,26,27] and miRNAs [28]. These factors contribute in various ways to shifting the immune response and protecting the parasite. *Fh*-FABPs induce a tolerogenic phenotype in human dendritic cells [29] and suppress the expression of proinflammatory factors (IL-1β and tumor necrosis factor alpha (TNF-α)) in monocytes [30], similar to activin/TGF-like molecule (*Fh*-TLM—a member of the TGF superfamily), which induces a Th_2_/T_reg_-like phenotype in macrophages [31]. Proteases may activate the NLR family pyrin domain containing 3 inflammasome (NLRP3) [32] and protect the parasite by cleaving antibodies. Antioxidants exhibit pleiotropic effects by directly promoting a Th_2_ response, preventing the development of a tissue-damaging Th_17_ response, and detoxifying host reactive metabolites [22]. It seems that an overall effect of *Fh*-ES is a combination of both redundant and non-redundant actions of its components on the immune system, and further characterization of this impact is required.

Macrophages are an important part of both the innate and adaptive immune responses, although their interaction with *Fh*-ES is still not fully understood. While data show a common trend—*Fh*-ES’s ability to skew the immune response toward Th_2_/T_reg_—the mechanisms may be species-dependent. Previously, we demonstrated that *Fh*-ES reduces TNF-α release from bovine macrophages [20,33]. A similar anti-inflammatory effect of *Fh*-ES (or its fractions) has been shown for human monocytes and mouse macrophages; however, different mechanisms were observed in these models. In mouse macrophages, *Fh*-ES increased the release of TGF-β and IL-10, indicating a hallmark of Th_2_ activation [34]. On the other hand, *Fh*-EVs (*Fasciola hepatica* Extracellular Vesicles) affected neither TNF-α mRNA expression nor its release by human monocytes. However, prolonged stimulation with LPS and *Fh*-EVs increased the mRNA expression of TGF-β and IL-13, powerful modulators of T_reg_ and Th_2_ responses, respectively [35]. The various mechanisms responsible for the upregulation of Th_2_/T_reg_ mediators and the downregulation of Th_1_/Th_17_ mediators remain to be elucidated. Resolving this puzzle would enhance our understanding of the molecular mechanisms occurring not only during fascioliasis but also in other helminthiases. Transcriptome profiling and biological big data analyses are undoubtedly powerful tools to decipher those interactions. miRNAs are potent regulators of gene expression and are heavily involved in regulating the immune system [36], including macrophage polarization [37]. These small, ~22 nt nucleotide-long molecules downregulate gene expression by binding to target mRNAs, leading to mRNA cleavage, destabilization, or inhibition of translation. The miRNAs impact on gene expression is challenging to decipher since a single miRNA can bind to hundreds of target mRNAs, and a single mRNA may be downregulated by multiple miRNAs [38]. Moreover, parasites have evolved the ability to manipulate the host miRNAome by transporting their own miRNAs into host macrophages [39]. Given the significant role of miRNAs in regulating macrophage phenotypes, we undertook a study to determine changes in miRNA expression profile in human macrophages. We chose a well-established cell line model—human THP-1 cells—which has been used in numerous parasitological experiments [40,41].

## 2. Materials and Methods

### 2.1. Fh-ES Preparation

*Fh*-ES collected from adult flukes (Weybridge strain) were generously provided by Dr. Luke Norbury (Witold Stefański Institute of Parasitology, Polish Academy of Sciences, Twarda 51/55, 00-818, Warsaw, Poland). Upon recovery of flukes from the rats’ bile ducts, the *Fh*-ES was collected as described previously [42] with slight modifications. The worms were incubated (37 °C, 5% CO_2_) in 40 mL RPMI1640 (Merck, Germany, Darmstadt) supplemented with penicillin (100 U/mL) and streptomycin (100 µg/mL) for 30 min to eliminate host tissue contamination and residues of bile. The medium was discarded and the flukes were rinsed twice with the prewarmed medium before adding 30 mL of fresh prewarmed medium. The worms were incubated for 36 h, with medium change every 90–120 min. Each batch was immediately frozen at −80 °C upon collection and stored until use. All batches were thawed and pooled together, and the protein concentration was measured using the Bradford assay. The *Fh*-ES was then concentrated using a 5 kDa cutoff Amicon filter (Merck, Darmstadt, Germany) as follows: 10 mL of *Fh*-ES was added on the membrane and centrifuged (3000× *g*, 4 °C) until volume reached 0.5 mL. The flow-through was discarded, and 12 mL of a fresh RPMI1640 medium (4 °C) without antibiotics was added. The sample was centrifuged again (3000× *g*, 4 °C) until the volume reached 0.5 mL. The cycle was repeated three times, resulting in a media exchange of 390,000×. The concentrated fraction was filtered through a 0.22 µm syringe filter, followed by protein concentration measurement using the Bradford method [43], and stored at −80 °C until use.

### 2.2. Assessment of Endotoxin Level

The endotoxin level was evaluated using the Pierce LAL (*Limulus amebocyte* lysate) Chromogenic Endotoxin Quantitation Kit (Thermo Fisher Scientific, Waltham, MA, USA).

### 2.3. SDS PAGE Analysis

The concentrated fraction (10 µg of *Fh*-ES) was subjected to SDS PAGE analysis. Briefly, 10.6 µL of concentrated *Fh*-ES (0.93 ng/µL) were mixed with 42.4 µL of phosphate-buffered saline (PBS) and 13.25 µL of 5 × SDS PAGE Dye (10% SDS, 5% β-mercaptoethanol, 50% Glycerol, 500 mM Tris-HCl (pH 6.8) and 0.05% bromophenol blue dye) followed by incubation at 95 °C for 5 min. The sample and the molecular weight marker (Thermo Fisher Scientific Cat. No. 26619) were loaded onto a 4% stacking gel and resolved (40 V) until they reached a 12.5% polyacrylamide resolving gel containing 0.2% SDS and further resolved (at 80 V) until the bromophenol blue reached the bottom of the gel. The gel was stained overnight with a staining solution (5% *v*/*v* acetic acid, 30% *v*/*v* methanol, 0.1% *w*/*v* Coomassie Brilliant Blue R250) with gentle shaking following destaining using destaining solution (5% *v*/*v* acetic acid, 30% *v*/*v* methanol). 

### 2.4. THP-1 Macrophages Stimulation

THP-1 monocytes were purchased from American Type Culture Collection (ATCC, Manassas, VA, USA). The monocytes were cultured at 37 °C with 5% CO_2_ in RPMI 1640 supplemented with 10% fetal bovine serum (FBS), 100 U/mL penicillin, and 100 µg/mL streptomycin. Once the cells reached the appropriate number, they were seeded at 5 × 10^5^ cells per well in a 24-well cell culture plate and differentiated into macrophages for 48 h using phorbol 12-myristate 13-acetate (PMA) (30 ng/mL). The cells were rinsed 3 times with fresh medium followed by treatment with either lipopolysaccharide (LPS) (100 ng/mL) for control cells or LPS (100 ng/mL) and *Fh*-ES (7 µg/mL) for experimental cells. Considering the final endotoxin level in the cell culture medium as an important parameter of the experiment, the concentration of *Fh*-ES used to stimulate THP-1 macrophages resulted in an endotoxin enrichment of the medium to a final concentration of 0.06 EU/mL, which complies with the strict FDA recommendations for endotoxin levels, even for solutions in contact with cerebrospinal fluid [44]. The cells were stimulated in octuplicates and incubated for 48 h (37 °C, 5% CO_2_). The medium was discarded followed by suspending the cells in QIAzol Lysis Reagent (Qiagen, Venlo, The Netherlands). The suspension of the lysed cells was stored until use at −80 °C.

### 2.5. Microarray Experiments

miRNA was isolated using the miRNeasy Mini Kit (Qiagen, Venlo, The Netherlands). The RNA concentration was assessed spectrophotometrically. The RNA quality and integrity were examined with the Agilent 2100 Bioanalyzer using Agilent RNA 6000 Nano Kit (Agilent Technologies, Santa Clara, CA, USA). The RNA integrity number (RIN) values for each sample were above 9. The RNA from each well (8 control wells and 8 experimental wells) was prepared for hybridization with arrays using the miRNA Complete Labeling and Hyb Kit (Agilent Technologies, Santa Clara, CA, USA) and hybridized to the Human miRNA Microarray, Release 21.0, 8 × 60 K (G4872A, Agilent Technologies) according to the manufacturer’s protocol. Microarrays were scanned using Agilent DNA Microarray Scanner G2505C and analysis of hybridization intensities was performed using Agilent Feature Extraction (FE) Software, version 10.7.3.1. The results were analyzed using GeneSpring 14 software (Agilent Technologies, Santa Clara, CA, USA).

### 2.6. Statistical Analyses

The analyses were performed on miRNA samples obtained from eight wells of control samples (LPS-activated) and eight wells of experimental samples (LPS-activated and *Fh*-ES). The probe sets were filtered by flags to remove poor-quality probes (absent flags) and were compared using a moderated t-test. However, as commonly observed, using a t-test to analyze multiple samples results in a number of false-positive results [45]. To address this issue, various multiple testing corrections were applied to adjust the *p*-values for multiple comparisons. These corrections included the Family-Wise Error Rate (FWER) methods: Bonferroni, Bonferroni-Holm, and Westfall-Young Permutation, and the False Discovery Rate (FDR) methods: Benjamini–Hochberg, Storey with Bootstrapping, and Storey with Curve Fitting. The corrections are listed in Table 1 according to their stringency [46], which defines their abilities to reduce the generation of false-positive data. The statistical analyses were performed using GenSpring 14 software (Agilent Technologies, Santa Clara, CA, USA).

## 3. Results

### 3.1. Fh-ES Assessment Using SDS-PAGE

**Figure 1:**
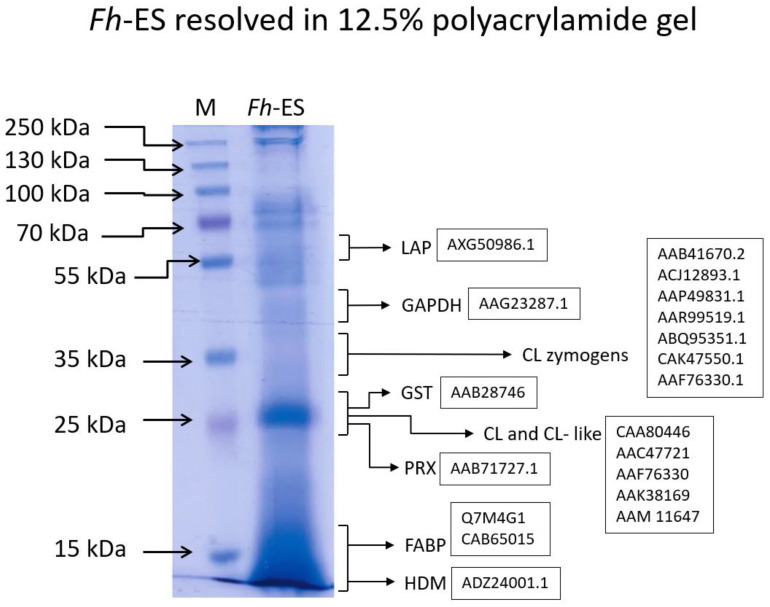
*Fh*-ES (10 µg) resolved in 12.5% polyacrylamide gel. M—molecular weight marker (ThermoFisher Scientific Cat. No. 26619), *Fh*-ES—*Fasciola hepatica* Excretory-Secretory Products. The protein identification in the bands is defined in the literature review. For the analyzed bands, the protein is given with Gen Bank numbers. CL—Cathepsin L, CL-like—Cathepsin L-like protein, FABP—fatty acid binding protein, GAPDH—glyceraldehyde-3-phosphate dehydrogenase, HDM—helminth defense molecule, LAP—leucine aminopeptidase, PRX—peroxiredoxin.

The *Fh*-ES were resolved in a polyacrylamide gel prior to cell stimulation. A specific pattern was obtained (Figure 1), and the particular bands were identified according to the literature, with an emphasis on their immunomodulatory functions. The ~25 kDa band represents the most abundant protein released by the adult fluke: *F. hepatica* Cathepsins L (CL) and Cathepsin L-like proteins (CL-like) [24,42] which are released as 30–38 kDa zymogens [47] and processed into their mature forms. CLs have been shown to induce NLRP3 inflammasome in dendritic cells [32] and modulate CD4 expression on T Cells [48]. Other components of the ~25 kDa band are likely to include *F. hepatica* peroxiredoxins (PRXs) [49] and *F. hepatica* glutathione S-transferases (GSTs) [50], both of which act as antioxidants [51] and immunomodulators, influencing macrophages. PRX induces alternative activation [26], and GST stimulates prostaglandin production by macrophages [50]. The other abundant fraction (~12 kDa) likely represents *F. hepatica* fatty acid binding proteins (FABPs) [51], which induce alternative activation of macrophages [52] and *F. hepatica* helminth defense molecules (HDMs) [24,53], which are able to block Toll-like receptor (TLR) activation through LPS binding [27]. The composition of the proteins in bands with higher molecular masses is more challenging to decipher; however, *Fh*-*ES* is known to contain leucine aminopeptidases (LAP) (~70 kDa) [54] and somatic antigens like glyceraldehyde-3-phosphate dehydrogenase (GAPDH) and actin [42], which may be shed from the tegument during in vitro culture. The resolved fraction may also host proteins such as superoxide dismutase, serum albumin precursor, regucalcin or transferrin [42].

### 3.2. Statistical Analyses of Changes in miRNAnome Expression

The obtained microarray raw data were deposited in the Gene Expression Omnibus (GEO) database under the number GSE277108 and subjected to statistical analyses. A number of statistical methods were used to analyze the results. First, the moderated t-test was applied to identify miRNAs with changed expression fold (*p* < 0.05). The analyses without a correction identified 7 down-regulated and 11 upregulated miRNAs (Table 2). Nevertheless, multisample analyses are prone to I-type mistakes (generating false positive results). The classical *p*-value concept is suitable for testing differences between two samples, expecting one false positive discovery per twenty analyses when set as 0.05. During microarray analyses, multiple comparisons are made: mean expression difference of miRNA_1_ between the control group (LPS-activated macrophages) and the treated group (control stimulated with *Fh*-ES) is tested using a t-test. Next, the same comparison is made for miRNA_2_, miRNA_3_, … miRNA_n_. This approach results in an increase of false positive results (type 1 error), e.g., analyzing the expression of 3000 miRNAs with constant expression among samples will result in randomly identifying, with the highest probability, 150 (5%) as having changed expression (false positive). Assuming a further 20 miRNAs are expressed differently among samples and, if we identify all of them (rather unrealistic), we will have only a small fraction of true positives among all the positive hits: 20/(150 + 20). To overcome this issue, a statistical measure family-wise error rate (FWER) [55] was introduced for appropriate control of *p*-values [56]. It is defined as the probability of getting at least one false positive hit among the series of comparisons and can be calculated as FWER = 1 − (1 − α)^n^, where “α” is defined as a *p*-value threshold (0.05) and “n” as a number of comparisons. This formula shows that in analysis, e.g., 3000 miRNAs, we will get 1 - (1 − 0.05)^3000^ = 1, leading to conclusion of almost certainly false positive results. Controlling FWER is very efficient; nevertheless, it usually results in a decrease in true positive detection [57]. Another way to address multi-hypothesis analyses is the use of a parameter termed the false discovery rate (FDR) [56]. It is defined as a ratio of false positive to all positive hits, e.g., if there are 150 false positive hits and 20 true positive hits, FDR is calculated according to the formula: 150/170 = 88.2%. Both FWER and FDR may be controlled by the appropriate adjustment, but controlling FDR is more suitable for analyzing microarray data [58] due to lower stringency (an ability to reject false positive hits). During the microarray analysis, controlling both FWER and FDR was applied here. We used various corrections based on several modifications ranging from low to high stringency (Table 1) to increase the credibility of the results. Only the applied Westfall and Young permutation correction (*p*-value adjusted < 0.05) showed differences in expression of only one miRNA, miR-1537p (Table 2), while other corrections, including Benjamini–Hochberg, Bonferroni FWER, Bonferroni-Holm FWER, Storey with Bootstrapping, and Storey with Curve Fitting, showed no change in any miRNA upon stimulation of LPS-activated macrophages with *Fh*-ES (Table 2). There are no clear guidelines for FDR value since various experiments may require different approaches, e.g., clinical trials may require lower FDR than in vitro experiments [58], although it will increase false negative results. For that reason, we used a range of corrections, yet only one found a positive hit, which was considered an artifact. 

## 4. Discussion

*F. hepatica* infestation is characterized by a dynamic immune response characterized by mixed Th_2_/Th_17_ type during the acute stage and T_reg_ during established infestation [11]. The first stage is associated with NEJs‘ migration towards the liver through the intestine and peritoneum [11]. The latter stage is reached upon parasite settlement in the bile ducts and the release of immunomodulatory molecules [22]. The mounting T_reg_ response during the adult stage is to prevent damage to host tissue induced by Th_1_ response and to preserve the comfort niche for the parasite suitable for survival and reproduction [59]. *Fh*-ES is a complicated mixture of antigens: antioxidants, FABPs, cysteine proteases, protease inhibitors, mucin-like peptides, TGF-β mimics, HDMs [22], and EVs carrying both proteins [24] and miRNAs [28] impacting host immune cells. A large body of research indicates the role of *Fh*-ES-derived molecules in suppressing inflammation in experimental models of autoimmune diseases: *Fh*-HDM-1 may ameliorate symptoms of type I diabetes or multiple sclerosis [60], GST attenuates septic shock in mice [41,61] whereas purified EVs are beneficial during ulcerative colitis [62]. These data encourage further investigation on immunomodulatory potential and molecular mechanisms induced in immune cells by *Fh*-ES. However, these experiments revealed the impact of *Fh*-ES-derived molecules, not the whole fraction of *Fh*-ES, which leaves a gap in the knowledge regarding the impact of the complete *Fh*-ES fraction on the immune response.

We chose to scrutinize *Fh*-ES’s impact on macrophages, which are a very plastic population that is involved not only in immune responses but also in wound healing, fibrosis, and neuroinflammation regulation [63]. Previous studies have shown significant effects of *Fh*-ES on these cells. For instance, they have been reported to affect LPS-activated human monocytes through TLR activation or deactivation [64] and to induce apoptosis in mouse peritoneal macrophages [65] or death of human hepatocytes [66]. Our previous research also demonstrated that *Fh*-ES ameliorates LPS-induced proinflammatory response in bovine macrophages [67] and indicated that this amelioration may depend on the isolate used for *Fh*-ES collection [33]. To further characterize the impact of *Fh*-ES on macrophages and their role in the modulation of immune response towards a Th_2_ phenotype, we used a widely employed laboratory model of LPS-activated THP-1 macrophages stimulated with the full *Fh*-ES fraction. Surprisingly, the experiments showed no change in the expression of miRNAs upon stimulation of LPS-activated THP-1 macrophages with *Fh*-ES. Despite employing various statistical corrections, only one out of six tests indicated a change. Moreover, according to the method, the expression of only one miRNA was changed, which seems to indicate a statistical error. On the other hand, Wang et al. (2021) recently indicated a change in the miRNA profile in goat PBMCs upon stimulation with *Fg*-ES (*Fasciola gigantica* ES) [68]. However, they used a significantly higher concentration of *Fg*-ES (80 µg) compared to our study (7 µg), which might explain the different cellular responses. Similarly, Guasconi et al. (2012) reported on an apoptotic impact of *Fh*-ESP on mouse macrophages (both ex vivo and in vitro) using *Fh*-ES in the concentration of 50 µg/mL [65]. In both the above-mentioned experiments, cells were not subjected to LPS activation. On the other hand, THP-1 cells stimulated with *Toxocara canis* ES at a concentration of 5 µg/mL showed significant changes in cytokine profile (both with and without LPS activation) [40]. THP-1 macrophages were also sensitive to 5 µg/mL of *Hd*-ES (*Hymenolepis diminuta* ES), showing changes in the gene expression profile [69].

Although specific data on the effective dose of Fh-ES required to change miRNA profile in THP-1 macrophages are lacking, there is evidence that recombinant antigens can alter cytokine expression. For example, recombinant Fg-Cyst (Fasciola gigantica type I cystatin) at a concentration of 5 µg/mL dampens IL-6, cyclooxygenase-2 (Cox-2), and inducible nitric oxide synthase iNOS [70] in LPS-activated THP-1 macrophages, which are controlled by miR-146a [71], miR29b [72] and miR-369-3p [73], respectively. However, this effect was observed only at the protein level, with no changes in the mRNA level encoding IL-6, Cox-2, and iNOS [70]. Similarly, Fasciola hepatica Fh-FABP-12 at 5 μg/mL decreased the mRNA expression of proinflammatory IL-12a, TNF-α, and IL-1β in LPS-activated human macrophages ex vivo [52]. Of course, direct comparison of concentrations of single recombinant antigens and the mixture of molecules such as Fh-ES is challenging. While a single antigen may be sufficient to bind to its receptor on a cell, concentrations in vivo are typically lower, and cells are not exposed to such high concentrations. Nevertheless, in vitro and ex vivo models may require a higher concentration of antigens due to a lack of interactions with other cells and extracellular matrix (ECM) rendering them less responsive to natural concentrations. Moreover, the use of recombinant proteins may require higher concentration than their in vivo counterparts due to differences in post-translational modifications, dampening their biological activity, especially when expressed in prokaryotic expression systems that lack a number of eukaryotic modifications. Extracellular vesicles (Fh-EV) are a significant component of Fh-ES. Sanchez-López demonstrated that a much lower concentration (10 µg/mL) of Fh-EV is sufficient to increase TGF-β and IL-13 expression and decrease IL-6, IL-1β, C-X-C motif ligand 8 (CXCL8), and nuclear factor kappa-light-chain-enhancer of activated B cells (NF-κB) expression in LPS-activated THP-1 cells. This effect was not observed in cells stimulated with Fh-EV depleted of various fractions [35]. This suggests that smaller concentrations may be effective in experiments since physiological outcomes result from complex interactions between Fh-ES and cell receptors, rather than the effects of single antigens alone. Another issue to consider is the sensitivity of the method to detect the changes of miRNA levels and the biological effects of their fluctuations. Recent research has shown that while COVID-19 patients exhibited altered miRNA expression profiles in peripheral blood, the most highly upregulated and downregulated miRNAs changed their levels by 1.6 and 2.3 times, respectively [74]. Such changes were detectable using a different technique, that is Next Generation Sequencing (NGS) performed on 14 samples. Moreover, although the LPS activation of macrophages is associated with higher fold change in miRNA levels [75], the vast majority of miRNAs remain similar across M1, M2a, M2b, and M2c macrophages [76], with only eight miRNAs showing distinct expression differences; however, at the same time some mRNAs may change expression up to 80 times [76]. This addresses the issue of statistical analyses of microarrays. It is more challenging to find differences when the change of miRNA expression is low. Moreover, multiple sample analyses of miRNA results [77] require correction, decreasing false positive rates [78]. We used a number of corrections with a workflow from the least to the most conservative. Of course, it cannot be excluded that false negative results did not occur here since none of the methods is 100% reliable [79]. Similar experiments were conducted with mouse RAW364-7 macrophages, where NGS analyses revealed that the cells upregulated and downregulated 16 miRNAs, with the greatest decrease and increase in expression being 3.01 and 10.18, respectively [80]. Our experiments used LPS-activated macrophages and employed microarray technology, which recent data suggest is less suitable for miRNAome analyses compared to NGS [81]. This might explain the difficulty in identifying differences with our approach, especially if the particular macrophage population shows stably expressed miRNAs [76]. 

## 5. Conclusions

*Fh*-ES is a powerful modulator of host immune response; however, no changes in miRNA expression in the macrophages have been noted in this study. The results may be due to subtle changes not detectable by the microarray technique and the statistical analysis workflow. A deeper investigation is still to be performed, possibly through more sensitive NGS techniques.

## Figures and Tables

**Table 1:** Applied Corrections for Multiple Comparisons. The corrections are ordered according to their stringency (S) in comparison to the Benjamini–Hochberg correction. A higher number indicates a higher stringency of the test, corresponding to a lower probability of generating false-positive results.

S	Description	Correction
1	Independence of *p*-values across genes is assumed.	Benjamini–Hochberg (FDR)
	Storey with Bootstrapping. Storey with Curve Fitting.	Benjamini–Hochberg refinements
2	Permutates all the genes at the same time, accounting for their dependence.	Westfall and Young (FWER)
3	A stepwise procedure. Tests each hypothesis in an ordered sequence, allowing one to accept or reject a hypothesis based on the previous step, with a focus on power and stringency.	Bonferroni-Holm (FWER)
4	A single step procedure where each p value is corrected independently.	Bonferroni (FWER)

**Table 2:** The miRNAs considered as significant (*p* < 0.05) to change the expression upon stimulation of LPS-activated THP-1 macrophages with *Fh*-ES using a moderated t-test and several corrections. First, the analyses were performed using a moderated t-test and number of miRNAs were identified (*p* < 0.05). During the next step, various corrections were used to eliminate false positive results. ns—non significant (*p* > 0.05).

miRNA	Change	Fold	Benjamini–Hochberg	Storey with Bootstrapping	Storey with Curve Fitting	Westfall and Young	Bonferroni-Holm FWER	Bonferroni FWER
			*p*-Value					
hsa-miR-4730	down	6.58	ns	ns	ns	ns	ns	ns
hsa-miR-4728-3p	down	5.72	ns	ns	ns	ns	ns	ns
hsa-miR-1910-5p	down	4.12	ns	ns	ns	ns	ns	ns
hsa-miR-6824-3p	down	3.31	ns	ns	ns	ns	ns	ns
hsa-miR-4741	down	3.14	ns	ns	ns	ns	ns	ns
hsa-miR-4462	down	3.1	ns	ns	ns	ns	ns	ns
hsa-miR-26a-1-3p	down	2.6	ns	ns	ns	ns	ns	ns
hsa-miR-324-3p	up	1.4	ns	ns	ns	ns	ns	ns
hsa-miR-19b-1-5p	up	3.31	ns	ns	ns	ns	ns	ns
hsa-miR-7152-3p	up	3.51	ns	ns	ns	ns	ns	ns
hsa-miR-6780a-5p	up	4	ns	ns	ns	ns	ns	ns
hsa-miR-219a-5	up	4.09	ns	ns	ns	ns	ns	ns
hsa-miR-6512-5p	up	4.32	ns	ns	ns	ns	ns	ns
hsa-miR-378a-5p	up	4.52	ns	ns	ns	ns	ns	ns
hsa-miR-34c-5p	up	4.57	ns	ns	ns	ns	ns	ns
hsa-miR-487b-3p	up	5.15	ns	ns	ns	ns	ns	ns
hsa-miR-4651	up	5.17	ns	ns	ns	ns	ns	ns
hsa-miR-1537-3p	up	7.1	ns	ns	ns	<0.05	ns	ns

## Data Availability

The raw data were deposited in GEO database (No.: GSE277108).

## Abbreviations and Acronyms

CL	Cathepsins L
CL-like	Cathepsin L-like proteins
Cox-2	cyclooxygenase-2
CXCL8	C-X-C motif ligand 8
DALY	Disability-Adjusted Life Years
ECM	extracellular matrix
FDR	false discovery rate
FWER	family wise error rate
FABP	fatty acid binding protein
FBS	Fetal Bovine Serum
GAPDH	glyceraldehyde-3-phosphate dehydrogenase
GEO	Gene Expression Omnibus
GST	glutathione S-transferases
HDM	helminth defense molecule
iNOS	inducible nitric oxide synthase
LAL	Limulus amebocyte lysate
LAP	leucine aminopeptidase
LPS	lipopolysaccharide
miRNA	micro RNA
NEJ	newly excysted juvenile
NF-κB	nuclear factor kappa-light-chain-enhancer of activated B cells
NGS	Next-Generation Sequencing
NLRP3	family pyrin domain containing 3 inflammasome
PBS	phosphate-buffered saline
PRX	peroxiredoxin
TGF-β	transforming growth factor beta
TLR	Toll-like receptor
